# The acute effects of adjuvant radiation and chemotherapy on peripheral blood epigenetic age in early stage breast cancer patients

**DOI:** 10.1038/s41523-020-0161-3

**Published:** 2020-06-12

**Authors:** Mary E. Sehl, Judith E. Carroll, Steve Horvath, Julienne E. Bower

**Affiliations:** 1 Division of Hematology-Oncology, Department of Medicine, David Geffen School of Medicine, Los Angeles, CA USA; 2 Department of Biomathematics, David Geffen School of Medicine, Los Angeles, CA USA; 3 UCLA Jonsson Comprehensive Cancer Center, Los Angeles, CA USA; 4 Department of Psychiatry and Biobehavioral Sciences, David Geffen School of Medicine, Los Angeles, CA USA; 5 Cousins Center for Psychoneuroimmunology, UCLA Semel Institute for Neuroscience and Human Behavior, Los Angeles, CA USA; 6 Department of Human Genetics, David Geffen School of Medicine, Los Angeles, CA USA; 7 Department of Biostatistics, Fielding School of Public Health, Los Angeles, CA USA; 8 Department of Psychology, University of California, Los Angeles, CA 90095 USA

**Keywords:** Breast cancer, Epigenetics, Senescence

## Abstract

Survival has increased in early stage breast cancer (BC), and the late effects of treatment persist for decades. Molecular mechanisms underlying the acceleration of age-related diseases after chemotherapy and radiotherapy are poorly understood. We examined epigenetic changes in peripheral whole blood cells in early stage BC patients undergoing surgery followed by adjuvant radiotherapy, or surgery followed by adjuvant chemotherapy and radiotherapy. DNA methylation experiments were performed on whole blood samples collected before and after adjuvant therapy. Methylation profiles were used to estimate four measures of epigenetic age acceleration—intrinsic, extrinsic, phenotypic, and Grim—and cell counts. We found significant increases in extrinsic, phenotypic, and Grim epigenetic age acceleration and in estimated proportions of senescent T lymphocytes from pre- to post-treatment. When examining differential effects by treatment category, most of these increases were significant only in women undergoing radiation alone. Further studies are needed to examine whether these effects are related to the risk of cognitive and functional decline in BC survivors.

While multimodality cancer treatments prolong life in early stage breast cancer (BC) patients, they increase the risk for age-related health problems^1–6^, functional decline, and fatigue that profoundly impact the quality of life^7,8^. These secondary problems may be a consequence of accelerated biological aging by exposure to cancer therapies. However, although animal studies demonstrate that chemotherapy and radiation therapy induce cellular senescence^9–13^, relatively few studies have examined these effects in clinical cohorts^14–18^, and none have examined effects on epigenetic aging markers.

A promising biomarker of organism aging, the epigenetic clock, measures tissue age based on methylation levels of CpGs co-locating with genes underlying pathways associated with cell survival and self-renewal^19,20^. Estimated epigenetic age is tightly correlated with chronologic age, accelerated in disease states, and predictive of frailty and mortality^21–30^. Here we examine whether markers of epigenetic age acceleration increase following two common treatment regimens for women with early-stage BC: radiation and chemotherapy.

Patients were recruited from oncology practices in Los Angeles to participate in a longitudinal study of cancer-related fatigue^31^. Women were eligible for the parent study if they had been recently diagnosed with Stage 0–IIIA BC and had not yet started adjuvant or neoadjuvant therapy with radiation, chemotherapy, or endocrine therapy. Assessments were conducted before the onset of adjuvant therapy, after completion of radiation and/or chemotherapy, and over an 18-month follow-up (see Supplementary Fig. 1). The current analysis focuses on a subset of women (*n* = 72) who had blood samples available for epigenetic analyses at baseline and post-treatment. We selected women treated with radiation alone (*n* = 37) and women treated with chemotherapy followed by radiation (*n* = 35) to evaluate individual and combined effects of those treatment exposures. All women had completed surgery prior to the baseline assessment.

We examined four measures of epigenetic age acceleration: intrinsic (IEAA), extrinsic (EEAA), phenotypic (PEAA), and Grim (GEAA), based on weighted averages of methylation levels at 353, 71, 513, and 1030 CpGs, respectively, with adjustment for chronologic age. Details of the epigenetic clock, DNA extraction/methylation experiments, and statistical analyses are provided in refs. ^19,20,25,26,32^ and Supplementary Methods. Briefly, while IEAA captures epigenetic age acceleration independent of changes in cell distribution that occur with advancing age, both EEAA and PEAA capture the functional decline of the immune system and exhibit negative and positive correlations with naive and late differentiated/senescent cytotoxic T lymphocytes, respectively. PEAA is also highly correlated with age-related phenotypes^32^. GEAA is strongly predictive of lifespan^33^. We estimated blood cell proportions using the advanced analysis option of the epigenetic clock software^19^ available online (http://dnamage.genetics.ucla.edu), which estimates the percentage of late differentiated CD8+ T cells (CD8+CD28−CD45RA−) and the number (count) of naive T cells (CD8+CD45RA+CCR7+). We examine changes in each of these measures from pre- to post-treatment, adjusting for ethnicity, body mass index (BMI), and tumor characteristics (ER, PR, and HER2 status, and stage), given links with epigenetic aging markers^21,34^.

Table 1 shows patient demographic, tumor, and treatment characteristics. A large percentage of women undergoing radiotherapy alone had ER+ and PR+ tumors and underwent lumpectomy rather than mastectomy. Women undergoing chemotherapy and radiotherapy had higher stage disease. There were no significant group differences in age, ethnicity, or BMI.

**Table 1:** Characteristics of the study sample.

	Radiation therapy alone (*N* = 37)	Chemotherapy and radiotherapy (*N* = 35)	Total (*N* = 72)	*p*-Value^a^
*Demographic characteristics*				
Age (years), mean ± SD	57.3 ± 9.3	56.1 ± 11.2	56.7 ± 10.2	0.44
Ethnicity, *N* (%)				
Hispanic	5 (14)	2 (6)	7 (10)	0.47
Non-Hispanic	32 (86)	33 (94)	65 (90)	
Education, *N* (%)				
HS degree	8 (22)	8 (23)	16 (22)	0.41
College degree	12 (43)	16 (46)	28 (39)	
Postgraduate degree	17 (46)	11 (31)	28 (39)	
Body mass index, mean ± SD	25.6 ± 5.3	25.7 ± 6.7	25.7 (6.0)	0.96
Tobacco smoking, *N* (%)				
Current	1 (3)	1 (3)	2 (3)	0.59
Former	11 (30)	7 (20)	18 (25)	
Never	24 (65)	27 (77)	51 (71)	
Menopausal status, *N* (%)				
Pre-menopausal	8 (22)	9 (26)	17 (24)	0.64
Peri-menopausal	1 (3)	3 (8)	4 (5)	
Post-menopausal	26 (70)	22 (63)	48 (67)	
Hysterectomy	2 (5)	1 (3)	3 (4)	
*Tumor characteristics*				
ER positive, *N* (%)	36 (97)	26 (74)	62 (86)	**0.013**
PR positive, *N* (%)	32 (86)	21 (60)	53 (7)	**0.023**
HER2 amplified, *N* (%)	2 (5)	2 (6)	4 (5)	0.95
Stage, *N* (%)				
0	5 (13)	0 (0)	5 (7)	**0.00015**
1	25 (68)	11 (31)	36 (50)	
2	7 (19)	18 (51)	25 (35)	
3	0 (0)	5 (14)	5 (7)	
Type of surgery				
Lumpectomy	35 (94)	27 (77)	62 (86)	**0.032**
Mastectomy	2 (5)	8 (23)	10 (14)	

^a^*p*-Value comparing treatment groups.

*p*-values were in bold if < 0.05.

Predicted epigenetic age (DNAm age) was significantly correlated with chronologic age for IEAA (*r* = 0.85, *p* < 0.00001), EEAA (*r* = 0.8, *p* < 0.00001), PEAA (*r* = 0.8, *p* < 0.00001), and GEAA (*r* = 0.89, *p* < 0.00001) in pre-treatment samples from the full cohort. Corresponding age acceleration measures for each of these epigenetic biomarkers are defined as the residuals from regressing DNAm age on chronologic age, and are measured in years.

Figure 1 shows box pots of age acceleration measures from pre- to post-treatment for the full sample; plots for the two treatment groups are shown in Supplementary Fig. 2.

**Fig. 1:**
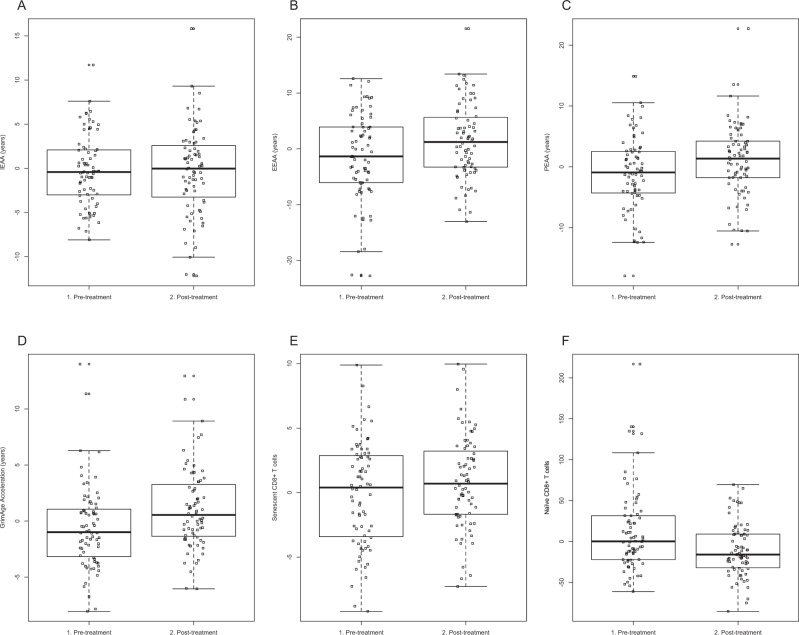
Full distributions for measures of age acceleration in peripheral blood from pre- to post-treatment. In addition to intrinsic (IEAA, **a**), extrinsic (EEAA, **b**), phenotypic (PEAA, **c**), and Grim (GEAA, **d**) measures of age-acceleration, age-adjusted estimates of senescent cytotoxic T lymphocytes (**e**) and naive T lymphocytes (**f**) are shown for the full sample. Our repeated measures ANOVA analysis revealed significant increases in EEAA (*p* = 0.0021), PEAA (*p* = 0.015), GEAA (*P* = 3.2 × 10^−6^), and age-adjusted estimates of senescent cytotoxic T lymphocytes (*p* = 0.038). Age-adjusted estimates of naive T lymphocytes decreased after treatment (*p* = 2.2 × 10^−5^). There was no significant change in IEAA with treatment (*p* = 0.83). Supplementary Fig. 2 reveals boxplots for each of these age acceleration measures and estimated cell counts, separated by treatment type.

In the full sample receiving adjuvant treatment, repeated measures analysis of variance showed a significant increase in EEAA (*F*(1,71) = 10.2, *p* = 0.0021), PEAA (*F*(1,71) = 6.22, *p* = 0.015), and GEAA (*F*(1,71) = 25.6, *p* = 3.2 × 10^−6^) from pre- to post-treatment, adjusting for ethnicity, BMI, stage, and ER/PR/HER2 status. Amongst patients receiving radiotherapy alone, EEAA (*F*(1,36) = 16.0, *p* = 3.0 × 10^−4^), PEAA (*F*(1,36) = 5.94, *p* = 0.020), and GEAA (*F*(1,36) = 11.7, *p* = 0.0015) were significantly increased, but not IEAA. Patients receiving both chemotherapy and radiation exhibited a significant increase in GEAA (*F*(1,34) = 13.6, *p* = 8.0 × 10^−4^), and non-significant increases in EEAA (*F*(1,34) = 0.66, *p* = 0.42) and PEAA (*F*(1,34) = 0.87, *p* = 0.36). There was a notable decrease (of borderline significance) from pre- to post-treatment in IEAA in the group receiving both chemotherapy and radiotherapy (*F* = 3.68, *p* = 0.064), and this finding is consistent with a recent report of decreased DNAm age with G-CSF administration^35^.

The proportion of late differentiated/senescent T lymphocytes increased after treatment in the full sample (Fig. 1e) (*F*(1,71) = 4.5, *p* = 0.038), and in the group treated with radiotherapy alone (Supplementary Fig. 2e) (*F*(1,36) = 7.7, *p* = 0.0077). There was an accompanying decrease in naive T lymphocytes in the full sample (*F*(1,71) = 20.7, *p* = 2.2 × 10^−5^) (Fig. 1f), and in both treatment groups (*F*(1,36) = 14.8, *p* = 4.5 × 10^−4^) for radiotherapy alone; (*F*(1,34) = 6.1, *p* = 0.019 for chemotherapy plus radiotherapy) (Supplementary Fig. 2f).

Because epigenetic biomarkers are not independent, we adjusted for multiple testing using a Bonferroni correction of 0.05/2. Using this criterion we find that increases in EEAA, PEAA, and GEAA, and the decrease in estimated naive T lymphocytes remained significant in the full sample and in the group receiving radiotherapy alone, while the increase in estimated senescent T lymphocytes remained significant only in the group receiving radiotherapy alone, and GEAA and the decrease in estimated naive T lymphocytes remained significant in the group receiving chemotherapy and radiotherapy.

Using a biomarker of biologic aging in whole blood, we found evidence of accelerating aging and immunosenescence after adjuvant therapy in women with early stage BC. To contrast these changes with “normal” aging, we examined age acceleration patterns in women of similar age but without cancer using published longitudinal data on DNA methylation studies of peripheral blood mononuclear cells of healthy women^36^. All women in the healthy cohort had at least two assessments, and the length of time between first and second visits ranged from 2 to 7 years. In this group, we found no significant changes from baseline to follow up in EEAA (mean 0.37 to −0.091, *p* = 0.35), IEAA (mean 0.0124–0.0115, *p* = 0.99), PEAA (0.90 to −0.30, *p* = 0.051), or GEAA (−0.063 to 0.018, *p* = 0.29). We also considered the possibility that changes in aging markers in the women undergoing cancer therapy might be driven by stress associated with a cancer diagnosis and treatment, but controlling for scores on the Perceived Stress Scale (PSS) yielded comparable results.

Examination of specific treatment exposures in our sample suggests a specific role of radiation in accelerating aging, although additional work is needed to confirm differential treatment effects and investigate mechanisms underlying these effects. Our findings are consistent with reports showing that adjuvant treatment for early stage BC accelerates biologic aging, as measured by p16^*INK4A*^ expression in T lymphocytes^14^ and lower telomerase activity^15^. Because epigenetic aging biomarkers predict frailty and mortality^23,32^, our results raise concerns about treatment as a potential accelerator of these processes and highlight the importance of identifying strategies to prevent accelerated aging in cancer survivors.

Limitations of our study include the small sample size, and the inability to directly examine the effects of chemotherapy alone on epigenetic age. Further studies are needed to examine the acute effects of chemotherapy alone and to examine whether accelerations in DNAm age persist in the years following recovery from surgery, chemotherapy, and radiotherapy. Another important limitation of our study is our inability to disentangle the relative contributions of cancer diagnosis and surgery from the presumed effects of adjuvant therapies. Women undergoing surgery alone after early stage breast cancer diagnosis do not provide an adequate comparison group, as these patients tend to carry a Stage 0 (ductal carcinoma in situ) diagnosis, and undergo mastectomy more frequently. Future work will examine global methylation changes associated with radiation and/or chemotherapy, particularly within pathways related to DNA repair. However, we would not expect global hypo- or hyper-methylation to influence our finding of age-related methylation patterns, as these are based on weighted averages of a specific subset of CpGs. Our results raise questions about whether the observed acceleration in epigenetic age is associated with adverse outcomes and toxicities associated with combined treatment modalities in early stage breast cancer, and further work should test the predictive utility of the epigenetic clock for adverse late effects in longitudinal research designs. Ultimately this information could be used to estimate risks of late effects due to accelerated aging after treatment of early stage breast cancer.

## Methods

Patients recruited to this longitudinal study provided informed written consent. This study was approved by the UCLA Institutional Review Board.

### Tissue acquisition and processing

Genomic DNA was extracted from buffy coats of peripheral blood samples using the MIDI DNAEasy Blood and Tissue Kit for the QIASymphony automated extractor (Qiagen). Purified DNA was placed into 96-well plates and concentrated using a SpedVac, and suspended in AE buffer to a minimum of 100 ng/μL. DNA was quantified using the Invitrogen Quant-iT dsDNA Assay Kit, high sensitivity (Invitrogen).

### DNA methylation data pre-processing

Bisulfite conversion using the Zymo EZ DNA Methylation Kit (ZymoResearch, Orange, CA, USA) as well as subsequent hybridization of the Human Methylation 850K EPIC chip (Illumina, San Diego, CA), and scanning (iScan, Illumina) was performed by the UCLA Neuroscience Genomics Core facilities according to the manufacturer’s protocols by applying standard settings. DNA methylation levels (*β* values) were determined by calculating the ratio of intensities between methylated (signal A) and un-methylated (signal B) sites. Specifically, the *β* value was calculated from the intensity of the methylated (M corresponding to signal A) and un-methylated (U corresponding to signal B) sites, as the ratio of fluorescent signals *β* = max(M,0)/[max(M,0) + max(U,0) + 100]. Thus, *β* values range from 0 (completely un-methylated) to 1 (completely methylated). To impute missing *β* values, we used a Euclidean metric to find *k*-nearest neighbors and impute the missing elements by averaging non-missing elements of its neighbors, using the impute.knn function in R^37^. Quantile normalization was applied to the raw data, in order to detect and remove outliers, and with the goal of making data comparable to the training data of the epigenetic clock.

### Measures

Survey data were available from the RISE study on patients’ age, ethnicity, and education. BMI was determined through measurement of height and weight at the baseline assessment. Tumor characteristics and type of treatments received were determined by medical record review.

### Statistical methods and analysis

We used four well-established measures to estimate epigenetic age based on weighted averages of CpGs: intrinsic (353 CpGs^19,25^), extrinsic (71 CpGs^20,26^), phenotypic (513 CpGs^32^), and grim (1030 CpGs^33^). Residuals from linear regression of these measures on chronologic age are used to define the age-adjusted age acceleration measures: IEAA, EEAA, PEAA, and GEAA. Details of the epigenetic clock methodology are provided in the online clock software and tutorial^19,25^. Briefly, IEAA adjusts for imputed measures of blood cell counts and captures epigenetic age acceleration independent of cell distribution, while EEAA employs a weighted adjustment for the estimated blood cell counts from three blood cell types that change with age: naive (CD45RA+CCR7+) cytotoxic T lymphocytes (reflecting stem cell self-renewal), late differentiated/senescent (CD28−CD45RA−) cytotoxic T lymphocytes, and plasma B lymphocytes. PEAA is highly correlated with age-related phenotypes. Unlike IEAA, both EEAA and PEAA correlate with markers of immunosenescence, and both exhibit negative and positive correlations with naive and late differentiated/senescent cytotoxic T lymphocytes, respectively. Grim age is calculated using DNA methylation-based surrogate biomarkers of smoking pack-years and serum biomarkers known to be predictive of morbidity or mortality^33^. After regressing time-to-death on these DNAm-based biomarkers, the mortality risk estimate is transformed into an age estimate (Grim Age), and this age estimate is strongly predictive of lifespan^33^. We further estimated proportions of cytotoxic T lymphocytes in naive and senescent states using global methylation data, using the methods of Horvath^25^.

We used analysis of variance (ANOVA) with repeated measures to examine changes in four measures of age acceleration (IEAA, EEAA, PEAA, and GEAA) and estimated cell counts (naive and senescent cytotoxic T lymphocytes) from pre- to post-treatment, first combining across the two treatment groups, and subsequently examining each treatment group individually in order to assess whether the addition of chemotherapy further contributes to the effects of radiotherapy. All analyses were adjusted for ethnicity and BMI, given their known associations with epigenetic clock accelerations^34,38^. All analyses were also adjusted for tumor characteristics, including stage, and ER, PR, and HER2 status. Furthermore in order to examine for the effects of stress, we performed additional analyses adjusting for the PSS.

### Reporting summary

Further information on research design is available in the Nature Research Reporting Summary linked to this article.

## Supplementary information

Supplementary Information

Reporting Summary

## Data Availability

The data generated and analyzed during this study are described in the following data record: 10.6084/m9.figshare.11847369^39^. The methylation data discussed in this publication have been deposited in NCBI’s Gene Expression Omnibus^40^ and are accessible through GEO Series accession https://identifiers.org/geo:GSE140038 ^41^.
