# Supplementary material for: The acute effects of adjuvant radiation and chemotherapy on peripheral blood epigenetic age in early stage breast cancer patients
Source: NPJ Breast Cancer. 2020 Jun 12;6:23. doi: 10.1038/s41523-020-0161-3 (PMC7293278; doi:10.1038/s41523-020-0161-3)

Supplementary Figure 1. Timeline of treatment course and study visits by treatment group.

Group 1: Radiotherapy alone

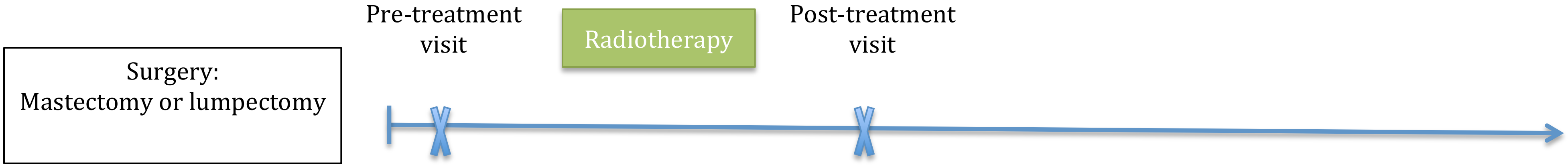

Group 2: Chemotherapy and radiotherapy

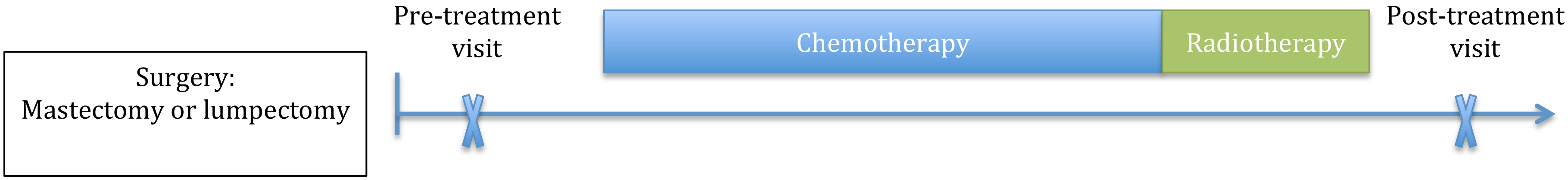

Supplementary Figure 2.

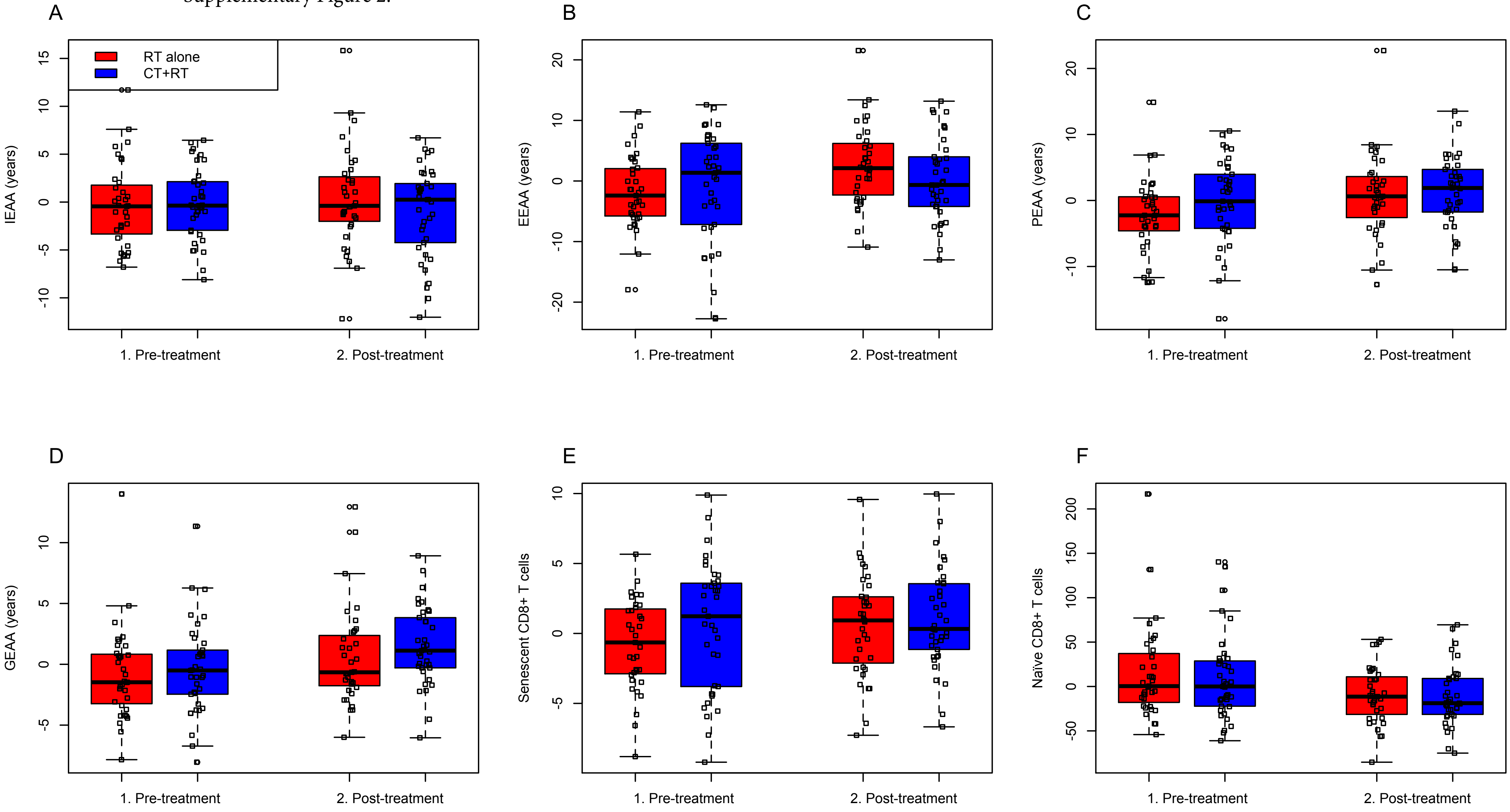

Supplement: Supplementary file 1 — Supplementary Information [file 41523_2020_161_MOESM1_ESM.pdf]
